# Sexual violence against women and girls in South Sudan

**DOI:** 10.1186/s13031-020-00268-y

**Published:** 2020-04-07

**Authors:** Dilshad Jaff

**Affiliations:** Maternal and Child Health Department, Gillings School of Global Public Health, University of North Carolina at Chapel Hill, 135 Dauer Drive, Chapel Hill, NC 27599 USA

**Keywords:** Sexual violence, Armed conflicts, Complex humanitarian emergencies, Do no harm

## Abstract

This Letter to the Editor is in reference to the article by Murphy M, Ellsberg M and Contreras-Urbina M, “Nowhere to go: disclosure and help-seeking behaviors for survivors of violence against women and girls in South Sudan,” published on 12 February 2020. The authors have to be lauded to study this important topic in South Sudan where data are scarce and the problem is less understood. In such a context, actions by various actors to address sexual violence, a major public health concern and a serious international humanitarian law and human rights violation, must be well thought of to avoid causing more harm and compound the suffering of survivors.

## Main text

I found the recent research on disclosure and help seeking behaviors for survivors of violence against women and girls in South Sudan by Murphy M, Ellsberg M and Contreras-Urbina M [1], a praiseworthy contribution to the literature.

Sexual violence, a major public health concern and a serious international humanitarian law and human rights violation, has both short and long term physical and mental consequences. Not only among women and girls, armed conflicts and complex humanitarian emergencies put men and boys within the population at a high risk. While addressing help seeking behaviors for survivors is important, ensuring the continuum of care is critical.

In South Sudan, the youngest country in the world, in addition to the lack of sustainable quality health services, data are scarce and the magnitude of the problem is not even known, much less understood. Therefore, designing and implementing evidence-based interventions tailored to needs in a timely manner is challenging. As a mechanism to overcome that, the International Committee of the Red Cross (ICRC) applies a ‘reverse burden of proof’ in conflict zones, which presumes the occurrence of sexual violence unless proven otherwise. This is enabling the organization to start actions without any delay and ultimately creating more space for survivors to get the necessary help. It is noteworthy to mention that this approach is unique in responding to the needs of the victims within the humanitarian sphere.

It is important to ‘making the needs of the invisible, visible’ with keeping in mind the ‘Do No Harm’ principles to ensure that these actions don’t cause more harm and compound the suffering of survivors. Protecting the dignity and safety of civilians and victims of armed conflicts is critical. All concerned actors must ensure such practices.

## Data Availability

Not applicable.

## Abbreviation

ICRC: The International Committee of the Red Cross
